# Cost-effectiveness of psychological intervention within services for depression delivered by primary care workers in Nepal: economic evaluation of a randomized control trial

**DOI:** 10.1017/gmh.2022.54

**Published:** 2022-10-31

**Authors:** L. R. Aldridge, N. P. Luitel, M. J. D. Jordans, J. K. Bass, B. Patenaude

**Affiliations:** 1 Department of Mental Health, Johns Hopkins University Bloomberg School of Public Health, Baltimore, MD, USA; 2 Research Department, Transcultural Psychosocial Organization Nepal, Kathmandu, Nepal; 3 Center for Global Mental Health, IoPPN, King's College London, London, UK; 4 Department of International Health, Johns Hopkins University Bloomberg School of Public Health, Baltimore, MD, USA

**Keywords:** Cost-effectiveness analysis, depression, integration

## Abstract

**Background:**

Integrating services for depression into primary care is key to reducing the treatment gap in low- and middle-income countries. We examined the value of providing the Healthy Activity Programme (HAP), a behavioral activation psychological intervention, within services for depression delivered by primary care workers in Chitwan, Nepal using data from the Programme for Improving Mental Health Care.

**Methods:**

People diagnosed with depression were randomized to receive either standard treatment (ST), comprised of psychoeducation, antidepressant medication, and home-based follow up, or standard treatment plus psychological intervention (T + P). We estimated incremental costs and health effects of T + P compared to ST, with quality adjusted life years (QALYs) and depression symptom scores over 12 months as health effects. Nonparametric uncertainty analysis provided confidence intervals around each incremental effectiveness ratio (ICER); results are presented in 2020 international dollars.

**Results:**

Sixty participants received ST and 60 received T + P. Implementation costs (ST = $329, T + P = $617) were substantially higher than service delivery costs (ST = $18.7, T + P = $22.4) per participant. ST and T + P participants accrued 46.5 and 49.4 QALYs, respectively. The ICERs for T + P relative to ST were $4422 per QALY gained (95% confidence interval: $2484 to $9550) – slightly above the highly cost-effective threshold – and −$53.21 (95% confidence interval: −$105.8 to −$30.2) per unit change on the Patient Health Questionnaire.

**Conclusion:**

Providing HAP within integrated depression services in Chitwan was cost-effective, if not highly cost-effective. Efforts to scale up integrated services in Nepal and similar contexts should consider including evidence-based psychological interventions as a part of cost-effective mental healthcare for depression.

## Introduction

One in 27 people with depression receives minimally adequate mental healthcare in low- and lower-middle-income countries, compared to one in five in high-income countries (Thornicroft *et al*., 2017). Mental health accounts for less than two percent of total health spending in low- and lower-middle-income countries, despite depression alone being the second leading contributor to global disability (Patel *et al*., 2018; Vigo *et al*., 2019; GBD 2019 Mental Disorders Collaborators, 2022). Integrating mental health services into primary care and other service delivery platforms has been recognized and implemented as a key strategy for reducing the gap between burden and available mental healthcare in low- and middle-income countries (LMIC) (Collins *et al*., 2011; Patel *et al*., 2018; Kola *et al*., 2021). Integrated care models typically rely on task sharing approaches wherein non-specialist health workers, such as nurses and community health workers, are trained to identify people with mental disorders and provide treatment under specialist supervision (Beaglehole *et al*., 2008; Kakuma *et al*., 2011). In 2008, the World Health Organization established guidelines for the delivery and scale up of task sharing approaches for mental health through the Mental Health Gap Action Programme (mhGAP) (World Health Organization, 2008). Since then, these guidelines have been implemented as part of mental health and psychosocial support services in more than 90 countries (Keynejad *et al*., 2018). Evidence for mhGAP is part of a wider, robust evidence base developed over the past two decades on the effectiveness of task sharing interventions to improve a range of mental health conditions (van Ginneken *et al*., 2011; Singla *et al*., 2017; Hoeft *et al*., 2018; Karyotaki *et al*., 2022).

Task sharing and service integration approaches are based on the premise these models represent a more efficient, cost-effective allocation of limited resources compared to traditional models of centralized, specialist service delivery (Beaglehole *et al*., 2008). Despite this, there is a dearth of economic evidence supporting the use of these models to treat depression (Herrman *et al*., 2022) and other mental health disorders (Knapp and Wong, 2020). A 2020 systematic review by Cubillos and colleagues (2020) found strong evidence integrating mental health services into primary care is effective for reducing clinical symptoms and functional impairment of depression within LMIC. However, the authors identified only seven studies examining the cost-effectiveness of integrated services for treating depression, with three of these studies relying on modeling rather than experimental data.

Experimental data are necessary to build the economic evidence base, test integration assumptions, and establish standardized frameworks to guide economic research within global mental health (Cubillos *et al*., 2020). Economic evaluations based on prospective studies also provide valuable opportunities to cost resources required to implement mental health interventions. Implementation costs are often underestimated within public health research and can qualitatively affect the results of economic evaluation (Sohn *et al*., 2020). Incomplete cost data may stem from economic evaluations conducted *post hoc* where important cost drivers are not captured, resource requirements are underestimated, and other limitations arise common to retrospective data collection (Sohn *et al*., 2020). Some underestimated costs may also be a result of training approach. Researchers implementing global mental health interventions often rely on international experts to provide in-person training and oversee service implementation (Fairburn and Patel, 2014). These approaches can result in service delivery models that are not sustainable or scalable, particularly if these implementation costs are not accurately incorporated into economic evaluations. Accurate and comprehensive cost profiles are necessary to inform health officials and other decision-makers when integrating and scaling up of mental health services within primary care systems.

We used prospective data to evaluate the cost-effectiveness of a psychological intervention within a package of treatment for depression from one of the largest mhGAP-based studies within LMIC, the Programme for Improving Mental Health Care (PRIME) (Lund *et al*., 2012). In Nepal, the PRIME consortium implemented a district mental healthcare plan in the district of Chitwan during the implementation phase from 2013 to 2017 (Lund *et al*., 2012; Jordans *et al*., 2016, 2019b). To expand access to mental health care at the health facility level, primary care workers were trained to identify and deliver mental health services for depression and other priority mental disorders according to clinical decision-making guidelines in the mhGAP Intervention Guide (World Health Organization, 2014). PRIME researchers imbedded a randomized control trial within a cohort study of these services to evaluate the effectiveness of two packages of mhGAP-based care for people with depression: a standard package of psychoeducation, antidepressant medication when indicated, and home-based follow up, *versus* this package plus the Healthy Activity Programme (HAP), a behavioral activation based psychological intervention (Jordans *et al*., 2019a).

Evidence from the nested randomized trial indicated that, while clinical and functional outcomes improved for all service users with depression over time (Jordans *et al*., 2019b), the psychological intervention was likely the key determinant in treatment effectiveness (Jordans *et al*., 2019a, 2020a). People with depression who received services without HAP did not have significantly greater improvements in clinical or functional outcomes compared to individuals with subclinical depression who received care as usual (Jordans *et al*., 2020a). These findings align with evidence indicating psychological interventions are effective in LMIC (Cuijpers *et al*., 2018; Karyotaki *et al*., 2022) and may have more enduring effects than antidepressants alone for people with depression (Cuijpers *et al*., 2013). Despite this, there is evidence primary care providers in Nepal prefer pharmacotherapy to psychological or psychosocial interventions when treating depression (Bhardwaj *et al*., 2022).

Existing cost estimates for PRIME depression services rely on modeling studies or research in other contexts and have yet to be updated with empirical estimates from PRIME services. Prior to service implementation, Chisholm and colleagues projected the cost-per-case of treating depression within primary care facilities in Nepal to be $1.86 for basic psychoeducation, advice, and follow up; $29.63 for antidepressant medication; and $2.32 for individual psychosocial counseling in 2008 USD (Chisholm *et al*., 2016). Cost estimates for HAP differ considerably from similar research in Goa, India, where delivering HAP within primary care cost an average of $65.66 per service user in 2015 international dollars ($19.69 in 2015 USD) (Weobong *et al*., 2017; World Bank, 2022). Chisholm and colleagues (2016) also estimated per-capita spending for scaled-up services by multiplying absolute prevalence by coverage by per-case costs, resulting in a cost-per-capita of $0.67 per capita at 15% national coverage levels. Related research indicated per-capita spending on mental health would need increase to $1.27 for mhGAP-based services to meet national coverage targets (Chisholm *et al*., 2017). However, these estimates are based on projectd costs, focus on service delivery alone, and lack valuable information on implementation costs relevant to health decision-makers.

Using prospective trial data, our primary objective was to evaluate the cost-effectiveness of providing psychological intervention within a package of depression services delivered by primary care workers in Chitwan, Nepal. A secondary objective was to construct a cost profile of service implementation and delivery for the two service packages included in the trial. Given psychological intervention may be the key to effective care for depression in this context, our goal was to produce economic evidence on the value of including this intervention within mhGAP-based service models and ultimately inform efforts in Nepal and similar contexts to scale up integrated mental health services.

## Methods

### Design

Research assistants recruited trial participants by screening eligible service users at ten participating primary care facilities in Chitwan, Nepal. Eligibility criteria required participants be 16 or older (the majority age in Nepal), reside in the study area, speak Nepali, be willing and able to provide informed consent, and not already be receiving treatment for depression or other mental health conditions. After confirming eligibility, research assistants then administered the nine-item Patient Health Questionnaire (PHQ-9) (Kroenke *et al*., 2001) adapted and validated for use in Nepal (Kohrt *et al*., 2016). Female Community Volunteers also identified and referred individuals from the surrounding catchment areas to PRIME facilities for depression screening using a proactive community case detection tool (Subba *et al*., 2017), which has been found to increase help-seeking behavior for depression and other mental health disorders (Jordans *et al*., 2020c). Individuals who screened positive on the PHQ-9 or who were referred by community health workers received a clinical diagnostic interview by a trained primary care provider. Primary care providers also identified some individuals with depression during routine consultations who were not screened positive by research assistant or referred by community volunteers. Individuals diagnosed with depression during clinical interview were invited to participate in the randomized control trial. Research assistants collected sociodemographic information at baseline and administered a series of questionnaires at baseline and three- and twelve-month follow up. Further information on study design, PRIME, and program impact in Nepal are available elsewhere (Baron *et al*., 2018; Jordans *et al*., 2019a, 2019b, 2020b; Aldridge *et al*., 2020).

### Setting

PRIME researchers trained primary care providers at 10 primary care facilities in Chitwan, Nepal on the identification, management, and follow up of depression and three other priority mental disorders – alcohol use disorder, psychosis, and epilepsy – according to the mhGAP Intervention Guide (Lund *et al*., 2012; World Health Organization, 2014). Primary care providers then delivered mental healthcare at the 10 participating primary care facilities from 2014 to 2017, with some training and research coordination beginning in 2013. Prior to PRIME, mental health services were only available at regional hospitals and some private facilities in Chitwan. Five percent of community members in a representative survey conducted by PRIME researchers screened positive prior to program implementation, though less than one in ten who screened positive for depression had received treatment for depression the past 12 months (Luitel *et al*., 2017).

### Interventions

Eligible participants diagnosed with depression were enrolled into a randomized control trial comparing two packages of integrated services: standard treatment (ST), which followed the mhGAP Intervention Guide in recommending psychoeducation, psychosocial advice, follow up, and antidepressant medication when indicated, or standard treatment plus psychological intervention (T + P) (Jordans *et al*., 2019a). Individuals in the T + P group received HAP, a manualized psychological intervention delivered at primary care facilities over six to eight weekly sessions. HAP was initially developed in India using culturally-adapted elements of evidence-based mental health treatment approaches and has been demonstrated to be clinically and cost-effective for individuals with moderate to severe depression when delivered by primary care providers in India (Chowdhary *et al*., 2016; Patel *et al*., 2017; Weobong *et al*., 2017; Bhat *et al*., 2022). The intervention is primarily based on behavioral activation therapy and includes elements of behavioral assessment, problem solving, activating social networks, and activity structuring and scheduling. Sessions are categorized into three phases intended to orient the individual, deliver components, and sustain skills after treatment, with the treatment goals of improved mood, improved life context, and reduced life problems (Chowdhary *et al.*, 2016).

### Health effects

We used quality adjust life years (QALYs) as the primary measure of health effect in our cost-utility analysis. To estimate QALYs, we relied on methods by Lokkerbol *et al*. (2021) in which sociodemographic factors and eight items from the 12-item WHO Disability Assessment Schedule (WHODAS) (Üstün, 2010) are regressed onto a preference-weighted disability index created using a nationally representative survey from 14 countries (Üstün *et al*., 2003). Both a general and population-specific mapping functions are available within their methods. We chose the mapping function representative of an Indian population as most relevant to our study population in Nepal (Lokkerbol *et al*., 2021). An equation in the supplement indicates the weight placed on each WHODAS and sociodemographic item used to generate the disability index (online Supplementary Equation S1). The WHODAS has been adapted, validated, and used widely for mental health research in Nepal (Tol *et al*., 2007; Luitel *et al*., 2013; Bimali *et al*., 2018; Risal *et al*., 2021), though the disability mapping method has not been validated against other measures of health utility.

The method to estimate QALYs from the WHODAS builds on previous methods used in South Asia (Buttorff *et al*., 2012) and in other LMIC (McBain *et al*., 2016; Patel *et al*., 2017). Health status is calculated as one minus the resulting disability index; a health status of one represents full health and 0 represents death. QALYs are then calculated by plotting health status over time and measuring the area under the curve (Yeo *et al*., 2019). Given the 12-month study follow up, participants could have gained up to one QALY during the trial. Our secondary measure of health effects relied on PHQ-9 scores as a measure of depression symptoms within cost-effectiveness analysis (Kroenke *et al*., 2001).

Research assistants administered the WHODAS and PHQ-9 to participants at all three study timepoints: baseline, 3 months, and 12-months. The WHODAS is comprised of 12 items where respondents are asked to report functioning in the previous two weeks across six domains – cognition, mobility, self-care, social interactions, life activities, and participation – using a Likert response ranging from 0 (‘none’) to 4 (‘extreme’) (Üstün, 2010). The PHQ-9 is a common measure of depression symptoms where respondents report the frequency of depressive symptoms (0 **‘**Not at all**’**, 1 **‘**Several days**’**, 2 **‘**More than half the days**’**, 3 **‘**Nearly every day**’**) over the previous two weeks on nine items (Kroenke *et al*., 2001). PHQ-9 responses are summed to produce a measure of symptom severity ranging from 0 to 27. Previous validation research has found a cutoff score of 10 to be indicative of depression in Chitwan with 94% sensitivity and 80% specificity and an internal consistency of *α* = 0.84 (Kohrt *et al*., 2016).

### Costs

Implementation costs for provider training and supervision were abstracted from PRIME administrative data and allocated according to resource use for each intervention under a five-year time horizon. Physical and mental health service delivery costs were calculated by multiplying healthcare use, reported by participants using the Client Socio-Demographic and Service Receipt Inventory (Chisholm *et al*., 2000) (online Supplementary Table S4), with the unit costs of healthcare resources (Stenberg *et al*., 2018) (online Supplementary Table S5). A detailed analysis of differences in healthcare use and costs across study groups are presented in a forthcoming paper. All cost data are reported in 2020 international dollars for the year 2020 from the societal perspective. Additional details of costing methods are presented in the supplementary materials.

#### Cost-effectiveness analysis

We compared the total cost of providing each treatment with the total health effects accrued by participants in each treatment group in our economic evaluation (Drummond *et al*., 2015). Our primary measure of cost-effectiveness is the incremental cost-effectiveness ratio (ICER), estimated by dividing the incremental cost of T + P over the incremental health effects of T + P relative to ST. We also present a secondary ICER based the incremental change in PHQ-9 scores as the measure of health effect. In addition to the program total approach to cost-effectiveness analysis presented in the main text, we also present an average approach to calculating ICERs in the supplementary materials that relies on the difference in median per-participant costs compared with difference in mean health effects per participant (online Supplementary Methods). Missing data for all costs and health effects were imputed using the multiple imputation chained equation (i.e. ‘mice’) package in R with predictive mean matching and 20 imputations.

Lastly, we used nonparametric bootstrapping methods to estimate a 95% confidence interval around the resulting sample-based ICER (Sanders *et al*., 2016). The method involved generating 1000 bootstrapped replicates by sampling from study participants within each group with replacement, calculating the difference in costs and health effects for each replicate, and then taking the middle 95th percentile of the resulting ICER replicates (online Supplementary Equation S2). A notable advantage of this method is that it maintains the association between each participant's costs and health effects within the repeated sampling frame. We then plotted the ICER replicates and calculated the percentage of replicates under one- and three-times 2020 gross domestic product (GDP) per capita in Nepal, which represent historical thresholds for highly cost-effective and cost-effective interventions, respectively (Word Health Organization, 2001). Despite the limitations of thresholds based on GDP (Marseille *et al*., 2015; Bertram *et al*., 2016), we present these thresholds to promote comparability with existing economic evaluations and because established thresholds for Nepal are under debate. We also present a cost-effectiveness acceptability curve in the supplementary materials that indicates the likelihood T + P is cost-effective across a range of thresholds (online Supplementary Figure S3).

## Results

### Participants

One-hundred twenty people diagnosed by primary care workers with depression were enrolled and randomized to receive ST (*n* = 60) or T + P (*n* = 60). At baseline, average participant age was 43.5 (standard deviation = 13.4) among ST and 39.0 among T + P (standard deviation = 14.1). Most participant were female (ST = 88%, T + P = 82%), had a partner (ST = 88%, T + P = 75%), and were Hindu (ST = 85%, T + P = 85%) (Table 1). A larger proportion of participants in the T + P group had completed primary school or more (53%) compared to those in the ST group (28%). Nine (15%) ST participants and 14 (23%) T + P participants were lost to follow up throughout the trial, including eight participants who did not participate in any HAP sessions (online Supplementary Fig. S1). T + P participants completed an average of 3.8 (standard deviation = 2.0) sessions over study follow up, with 60% completing between three and five sessions. Missing data for those lost to follow up and one cost outlier were multiply imputed and included in the analysis.

**Table 1.** Sociodemographic characteristics of trial participants

	ST (*n* = 60)	T + P (*n* = 60)
Female	53 (88%)	49 (82%)
Age of participant, mean (s.d.)	43.5 (13.4)	39.0 (14.1)
Employed	17 (28%)	15 (26%)
Education level		
Uneducated/illiterate	22 (37%)	14 (23%)
Less than primary	21 (35%)	14 (23%)
Primary school and above	17 (28%)	32 (53%)
Marital status		
Single	1 (2%)	7 (12%)
Has a partner	53 (88%)	45 (75%)
Divorced/widowed	6 (10%)	8 (13%)
Religion		
Hindu	51 (85%)	51 (85%)
Buddhist	8 (13%)	4 (7%)
Christian	1 (2%)	5 (8%)
Caste		
Brahmin/Chhetri	21 (35%)	27 (45%)
Janajati	21 (35%)	14 (23%)
Dalit	15 (25%)	14 (23%)
Others	3 (5%)	5 (8%)

ST, standard treatment; T + P, treatment plus psychological intervention; s.d., standard deviation.

### Healthcare costs

Training and supervision activities took place at primary care facilities and in the community throughout the PRIME Nepal implementation phase. PRIME relied on both primary care providers and community health workers to deliver mental health services. Primary care workers, including adjunct counselors trained through PRIME, provided most mental healthcare received by service users. Community health workers conducted home-based visits to encourage treatment attendance and follow up among service users. Trainings in each setting often involved a mix of both health worker cadres. Total training and supervision costs per service user totaled to $329 and $617 for ST and T + P, respectively (Table 2). Costs for delivering mental and physical health services were substantially lower than implementation costs. Median mental health service delivery costs were slightly higher among T + P participants, while the median cost of physical healthcare among ST participants, which includes services provided by traditional and indigenous healers, was more than double that of T + P participants (Table 2). A detailed investigation of differences in health service use and costs are presented in a forthcoming paper.

**Table 2.** Median costs per service user (Int$2020)

	ST	T + P
PRIME provider training		
Primary care	111.03	304.68
Community	150.39	241.68
PRIME provider supervision		
Primary care	0.86	2.31
Community	66.55	68.20
Healthcare use		
Mental	18.73	22.38
Physical	43.28	17.53
All	86.26	49.88

PRIME, Programme for Improving Mental Health Care; ST, standard treatment, T + P, treatment plus psychological intervention.

### Health effects

**Fig. 1.**
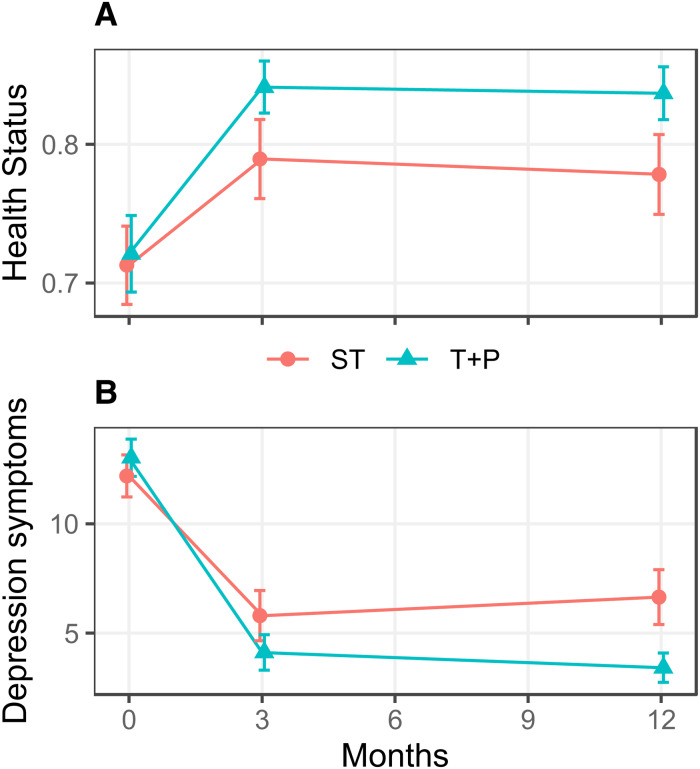
Health effects by group over follow up. (**A**) Change in health status over time. (**B**) Change in depression symptoms over time. ST, standard treatment; T + P, treatment plus psychological Intervention.

There were no significant differences between trial groups in health status (*p* = 0.21) or depression symptoms (p = 0.69) at baseline. Over 12-month follow up, ST participants gained a total of 46.5 QALYs and T + P participants gained 49.4, averaging 0.776 and 0.825 QALYs gained per participant for ST and T + P, respectively (Fig. 1, Table 2). Total depression symptom scores decreased (indicating improvement) by 332.2 points among ST participants and 576.0 among T + P from baseline to 12-month follow up, with an average change score per client of −5.54 (standard error  =  0.77) for ST and −9.60 (standard error  =  0.59) for T + P.

### Cost effectiveness

We combined total program costs for each treatment group with total health effects to estimate cost-effectiveness (Table 3). T + P cost $12973 more than ST, with 2.93 more QALYs gained and a 243.8-point greater decrease in depression scores among participants. This resulted in an ICER for T + P relative to ST of $4422 per QALY gained, which is slightly above the per capita GDP threshold of $4009 for being considered highly cost-effective and well under the cost-effectiveness threshold of three times per capita GDP. The ICER for changes in depression symptoms, our secondary measure of health effect, was $53.2 per point decrease on the PHQ-9. The per-client approach and a comparison of results for the total and per-client methods are presented in the supplement (online Supplementary Tables S1 and S2).

**Table 3.** Incremental cost-effectiveness of psychological intervention

			Health effects		ICER	
	*n*	Σ Cost	Σ QALYs	Σ(ΔPHQ-9)	QALYs	PHQ-9
ST	60	$29736	46.54	−332.15	–	–
T + P	60	$42709	49.47	−575.95	$4422	−$53.21

ST, standard treatment; T + P, Treatment + Psychological intervention; s.e., standard error; QALYs, quality adjusted life years; PHQ-9, Patient Health Questionnaire; ICER, incremental cost-effectiveness ratio.

**Fig. 2.**
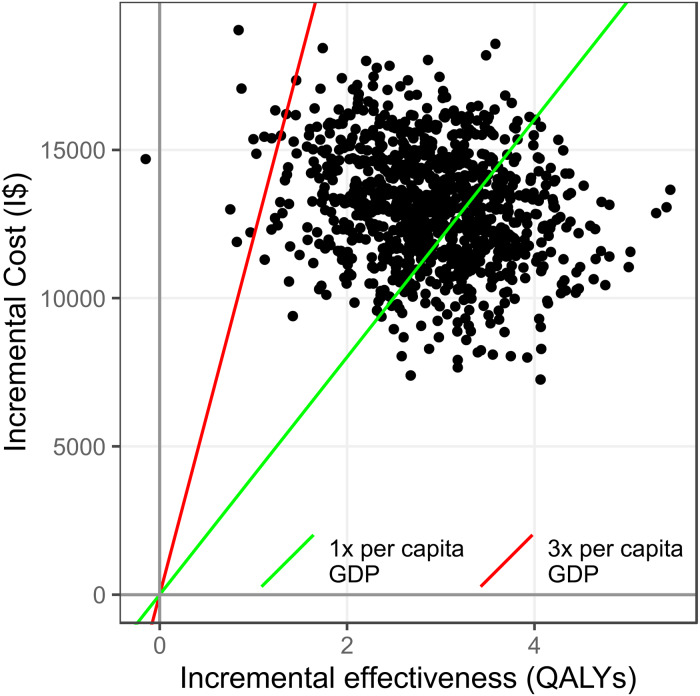
Nonparametric uncertainty analysis of cost-effectiveness. GDP Gross domestic product, I$ international dollars.

Nonparametric uncertainty analysis resulted in a 95% confidence interval of $2484 to $9550 per QALY gained. Approximately 37% and 99% of bootstrapped iterations were under the thresholds of one and three times gross domestic product per capita, respectively (Fig. 2). Uncertainty analysis for the secondary ICER resulted in a 95% confidence interval of −$105.8 to −$30.2 per unit change on the PHQ-9.

## Discussion

We conducted a cost-effectiveness analysis of providing a psychological intervention as part of primary-care-based services for people with depression in Chitwan, Nepal. Data for this study are from a randomized trial nested within PRIME between 2013 and 2017 of two mhGAP-based services packages: one with HAP, a behavioral activation psychological intervention, and another without. We found the cost-effectiveness of T + P to be $4422 per QALY gained in 2020 international dollars, well below the cost-effective threshold of three times per-capita GDP and slightly above the per capita GDP threshold for being highly cost-effective. Nonparametric uncertainty analysis indicated a very strong likelihood the ICER is below the cost-effective threshold, though a low likelihood it is below the highly cost-effective threshold. The findings and limitations of our study are considered within context of economic evaluation research in global mental health, including notable methodological approaches, before implications for practice and policy are presented.

A strength of our study is the prospective costing of implementation within cost-effectiveness analysis. Implementation costs are often underestimated during economic evaluations of public health interventions and can qualitatively affect research findings (Sohn *et al*., 2020). These costs, defined here as the economic value of resources used for provider training and supervision per client, were over ten times the cost of direct service delivery of PRIME services for depression. Though service delivery costs were similar to those projected during planning (Chisholm *et al*., 2016), failing to account for the cost of resources linked to their implementation would have substantively limited our understanding of the cost and value of both service packages. Implementation costs should be a key target when considering how to improve program efficiency.

A second strength is our use of novel methods to estimate individual health utility at each time point, rather than applying broad categories of generic disability weights for depression developed in other contexts. Methods published by Lokkerbol *et al*. (2021) enabled us to use sociodemographic factors and WHODAS items to calculate QALYs gained for each participant. WHODAS is a widely-used measure in global mental health with some evidence to indicate it is sensitive to changes in mental health and functioning (e.g., Habtamu *et al*., 2017). Notably, average baseline disability derived from WHODAS mapping is lower than the generic disability weight used by the Global Burden of Disease Studies for a moderate depressive episode (Global Burden of Disease Collaborative Network, 2020). In the absence of valid, sensitive quality of life measures appropriate for LMIC, deriving QALYs from WHODAS provides an opportunity to strengthen and standardize methodological approaches to economic evaluation within global mental health.

Our study is limited by the lack of established cost-effectiveness thresholds in Nepal. We refer to ‘historical’ cost-effectiveness thresholds based on per capita GDP initially developed by WHO (2001), which have been critiqued for their limited applicability to public health (Marseille *et al*., 2015; Bertram *et al*., 2016). More recent research has varied considerably in producing newer thresholds. Stenberg *et al*. (2014) estimate a threshold 1.5 to 2.0 times per capita GDP for low-income countries based on a review of evidence for the value of statistical life years. Woods *et al*. (2016) developed a threshold range of $109 to $1756 for Nepal, less than half of per capita GDP at its upper limit. Ochalek *et al*. (2018) estimated a threshold range of $256 to $291 for Nepal, approximately 6% of per capita GDP. We present our results according to the most commonly used, while flawed, cost-effectiveness thresholds from the WHO while also providing a supplemental figure (online Supplementary Fig. S3) to indicate the likelihood of cost-effectiveness across a range of alternate thresholds.

Our study is also somewhat limited in the scope of the societal perspective chosen for cost-effectiveness analysis. We account for all formal and informal healthcare costs in the societal perspective, such as transportation fees and the value of service user time (Sanders *et al*., 2016). However, we did not account for potential differences in the value of non-health related benefits. As such, there may be relevant differences in productivity, use of social services, or other non-health benefits not captured in our costing approach. Moreover, we present only the societal perspective comprised of formal and informal healthcare costs and do present the healthcare sector or payer perspectives. Given the vast majority of costs were incurred for training and supervision within the formal health system, it is highly unlikely adopting a more narrow perspective of the healthcare sector or payer would qualitatively affect our findings.

Our results align with similar research on HAP delivered within primary care in Goa, India. An economic evaluation of HAP in Goa, where the program was first developed, resulted in an ICER of $9333 per QALY gained compared to usual care, or roughly 60% of the state's per capita GDP but above national per capita GDP (Patel *et al*., 2017; Weobong *et al*., 2017). Recent research supports the long-term effectiveness and cost-effectiveness of HAP in India at five-year follow up (Bhat *et al*., 2022). A similar trial in Goa found collaborative stepped care compared to usual care for common mental disorders in primary care to be more costly in private facilities, cost saving in public facilities, and slightly more effective in both facility types (Buttorff *et al*., 2012). Overall, our findings generally align with the small evidence base for the cost-effectiveness of integrated services for depression in primary care within LMIC (Cubillos *et al*., 2020). A distinction of the present study is our inclusion of an active comparison group rather than comparing to usual care. Usual care, even ‘enhanced’ usual care, within mental health studies in LMIC typically means little to no actual treatment for depression is provided. Our findings indicate including a psychological intervention is cost-effective *above and beyond* active treatment delivered according to mhGAP guidelines.

The Ministry of Health in Nepal has adopted plans to scale up a model of services for priority mental disorders based on the evidence established by PRIME (Luitel *et al*., 2020). Future research should examine how the cost-effectiveness of the psychological intervention may change based on their model at scale. High implementation costs observed in the present trial will likely be reduced given economies of scale and changes to implementation strategies by the ministry, such as changes to who provides treatment, who conducts trainings, and the number of providers in each training cohort. Future research should also consider the equity of health and financial benefits conferred by integrated services for depression, as the present study was not sufficiently powered for subgroup analysis across key socioeconomic factors. Lastly, methods for economic research within global mental health should be standardized to improve the comparability and utility of findings. This includes building consensus for cost-effectiveness thresholds reflecting the willingness to pay for health spending in Nepal, rather than applying a generic GDP-based approach to all LMIC. Health utility measurement should also be standardized within mental health research given debate on the sensitivity of popular quality of life measures for mental health (Connell *et al*., 2014; Mukuria *et al*., 2016), and we support the use of WHODAS to estimate individual health utility rather than applying generic disability weights in the interim.

## Conclusion

Providing HAP is cost-effective within primary care services for depression in Chitwan, Nepal. Combined with previous evidence indicating psychological intervention is likely central to clinical and functional effectiveness, our findings provide further evidence that evidence-based psychological intervention should be included when integrating and scaling up services for depression in Nepal and similar contexts.

## Supplementary material

For supplementary material accompanying this paper visit https://doi.org/10.1017/gmh.2022.54. click here to view supplementary material
