# Supplementary material for: Cost-effectiveness of psychological intervention within services for depression delivered by primary care workers in Nepal: economic evaluation of a randomized control trial
Source: Glob Ment Health (Camb). 2022 Oct 31;9:499–507. doi: 10.1017/gmh.2022.54 (PMC9806977; doi:10.1017/gmh.2022.54)
Supplement: Supplementary file 1 [file S2054425122000541sup001.docx]

**SUPPLEMENTARY MATERIALS**

**Supplementary methods**

*Estimating implementation costs*

Implementation costs were abstracted from program financial reporting reports and categorized by (a) type, (b) treatment overlap, and (c) primary activity during the 2013 to 2017 implementation phase of PRIME Nepal. Cost type (a) refers to whether costs were financial, i.e., expenses directly incurred by the program, or economic, where the value of expenses not directly incurred by the program were estimated, such as health worker time spent in supervision. Treatment overlap (b) indicated whether costs were incurred for the delivery of HAP, depression-specific services included in basic mhGAP, or services overlapping depression and other priority mental disorders included in PRIME services. Lastly, the two categories of implementation activities (c) were the training and supervision of health workers.

Having compiled total program costs by the above categories, we then estimated implementation costs for all individuals who received services for depression under PRIME Nepal, including those not enrolled in the trial. Costs overlapping multiple disorders were allocated according to the proportion of patient volume across the ten primary care facilities during the implementation phase, after first confirming visit lengths were similar across disorders. Allocating according to patient volume resulted in 26.5% of shared implementation costs assigned to depression-based ST. Primary care providers received training in two types of psychological interventions – HAP for depression and Counseling of Alcohol Problems for alcohol use disorder – and costs shared across the two psychological interventions were split evenly. Lastly, we estimated financial and economic costs of ST and the incremental cost of HAP by dividing the sum of allocated costs for each intervention by the number of patients who received the intervention over the five-year implementation phase, resulting in implementation costs per-patient for ST and T+P.

*Estimating service delivery costs*

We also estimated the cost of providing health services for physical and mental health from a societal perspective **(Sanders et al., 2016),** which are presented in detail in a forthcoming paper. Briefly, service delivery costs were estimated by multiplying healthcare use with inflation-adjusted unit costs published by the World Health Organization (Chisholm et al., 2020; Stenberg et al., 2018; World Health Organization, 2009). Participants used the Client Socio-Demographic and Service Receipt Inventory (Chisholm et al., 2000) adapted for use in South Asia (Patel et al., 2007) to report health service use and out-of-pocket expenditure for formal and informal healthcare. Lastly, patient opportunity costs for attending health services were estimated by applying average participant income to time spent transporting to and from, waiting for, and receiving health services. Health service use was categorized as mental healthcare if the participant’s primary concern for symptoms or management of mental, neurological, or substance use conditions or if the visit were to a mental health specialist provider visit. Research assistants administered the Client Socio-Demographic and Service Receipt Inventory at all three time points, with each administration covering healthcare use over the previous three months. All cost data are reported in international dollars for the year 2020 (1 USD = 3.53 international dollars), the most recent year for which the conversion index based on purchasing power parity was available from the World Bank at the time of writing (World Bank, 2022b).

**Supplementary equations**

**Equation S1.** Deriving disability weights from WHODAS items and sociodemographic factors

disability weight = 0.0882 + (0.0158*S2) + (0*S3) + (0.0149*S4) + (0.0402*S6) + (0.0303*S8) + (0.0206*S9) + (0.0070*S11) + (0.0210*S12) + (0.0004*age) + (-0.0016*male) + (0.0002*single) + (-0.0001*married)

**Equation S2. Nonparametric uncertainty analysis of the incrementation cost-effectiveness ratio**

$$\hat{R}_{b}^{*}=\frac{\bar{C}_{T+P}^{*}-\bar{C}_{\mathrm{ST}}^{*}}{\bar{E}_{T+P}^{*}- \bar{E}_{\mathrm{ST}}^{*}}$$

$\hat{R}_{b}^{*}$= bootstrapped replicate of the ICUR, $\bar{C}^{*}$ = average cost for bootstrapped sample, $\bar{E}^{*}$ = average health effect in QALYS gained or change in PHQ-9 score for bootstrapped sample.

**Supplementary tables**

**Table S1.** Incremental cost-utility of psychological intervention (per client)

|  |  |  | **Health effect** | | **ICER** | |
| --- | --- | --- | --- | --- | --- | --- |
|  | n | Cost | QALYs (SE) | PHQ-9 (SE) | PHQ-9 | QALYs |
| ST | 60 | $412 | 0.776 (0.010) | -5.536 (0.768) | – | – |
| T+P | 60 | $663 | 0.825 (0.007) | -9.599 (0.581) | -61.95 | 5148 |
| ST Standard Treatment, T+P Treatment + Psychological intervention, SE standard error, QALYs quality adjusted life years, PHQ-9 Patient Health Questionnaire, ICER incremental cost-effectiveness ratio | | | | | | |

**Table S2.** Comparing results of total and per-client approaches to cost-effectiveness analysis

|  | ICER | 95% CI | < 1x pcGDP | < 3x pcGDP |
| --- | --- | --- | --- | --- |
| Per client | $5,148 | $3,330 to $10,151 | 16% | 99% |
| Total | $4,422 | $2,484 to $9,550 | 37% | 99% |
| ICER incremental cost-effectiveness ratio; pcGDP per capita gross domestic product; Results presented in 2020 international dollars | | | | |

**Table S3.** 2022 CHEERS Reporting Checklist

| **Section/topic** | **Item** | **Guidance for reporting** | **Page** |
| --- | --- | --- | --- |
| **Title** | | | |
| Title | 1 | Identify the study as an economic evaluation and specify the interventions being compared. | 1 |
| **Abstract** | | | |
| Abstract | 2 | Provide a structured summary that highlights context, key methods, results, and alternative analyses. | 2 |
| **Introduction** | | | |
| Background and objectives | 3 | Give the context for the study, the study question, and its practical relevance for decision making in policy or practice. | 4-7 |
| **Methods** | | | |
| Health economic analysis plan | 4 | Indicate whether a health economic analysis plan was developed and where available. | n/a |
| Study population | 5 | Describe characteristics of the study population (such as age range, demographics, socioeconomic, or clinical characteristics). | 11-12 |
| Setting and location | 6 | Provide relevant contextual information that may influence findings. | 8 |
| Comparators | 7 | Describe the interventions or strategies being compared and why chosen. | 8-9 |
| Perspective | 8 | State the perspective(s) adopted by the study and why chosen. | 10 |
| Time horizon | 9 | State the time horizon for the study and why appropriate. | 8 |
| Discount rate | 10 | Report the discount rate(s) and reason chosen. | n/a |
| Selection of outcomes | 11 | Describe what outcomes were used as the measure(s) of benefit(s) and harm(s). | 9-10 |
| Measurement of outcomes | 12 | Describe how outcomes used to capture benefit(s) and harm(s) were measured. | 9-10 |
| Valuation of outcomes | 13 | Describe the population and methods used to measure and value outcomes. | 9-10 |
| Measurement and valuation of resources and costs | 14 | Describe how costs were valued. | 10, Supplement |
| Currency, price date, and conversion | 15 | Report the dates of the estimated resource quantities and unit costs, plus the currency and year of conversion. | 10 |
| Rationale and description of model | 16 | If modelling is used, describe in detail and why used. Report if the model is publicly available and where it can be accessed. | n/a |
| Analytics and assumptions | 17 | Describe any methods for analysing or statistically transforming data, any extrapolation methods, and approaches for validating any model used. | 10-11 |
| Characterizing heterogeneity | 18 | Describe any methods used for estimating how the results of the study vary for subgroups. | n/a |
| Characterizing distributional effects | 19 | Describe how impacts are distributed across different individuals or adjustments made to reflect priority populations. | n/a |
| Characterizing uncertainty | 20 | Describe methods to characterise any sources of uncertainty in the analysis. | 11 |
| Approach to engagement with patients and others affected by the study | 21 | Describe any approaches to engage patients or service recipients, the general public, communities, or stakeholders (such as clinicians or payers) in the design of the study. | 8 |
| **Results** | | | |
| Study parameters | 22 | Report all analytic inputs (such as values, ranges, references) including uncertainty or distributional assumptions. | n/a |
| Summary of main results | 23 | Report the mean values for the main categories of costs and outcomes of interest and summarise them in the most appropriate overall measure. | 14 |
| Effect of uncertainty | 24 | Describe how uncertainty about analytic judgments, inputs, or projections affect findings. Report the effect of choice of discount rate and time horizon, if applicable. | 15 |
| Effect of engagement with patients and others affected by the study | 25 | Report on any difference patient/service recipient, general public, community, or stakeholder involvement made to the approach or findings of the study | 17 |
| **Discussion** | | | |
| Study findings, limitations, generalizability, and current knowledge | 26 | Report key findings, limitations, ethical or equity considerations not captured, and how these could affect patients, policy, or practice. | 15-18 |
| **Other relevant information** | | | |
| Source of funding | 27 | Describe how the study was funded and any role of the funder in the identification, design, conduct, and reporting of the analysis | 18-19 |
| Conflicts of interest | 28 | Report authors conflicts of interest according to journal or International Committee of Medical Journal Editors requirements. | 19 |

**Table S4.** Items from the adapted Client Socio-Demographic and Service Receipt Inventory

| **Health care use and costs** | | | | | |
| --- | --- | --- | --- | --- | --- |
| Instruction:  “*Excluding those times you may have been admitted for inpatient care, I want to know how many times you met any of the following health care providers in the past three months. That is, since [date].”* | | | | | |
| U1 | Traditional healer/spiritualist/herbalist |  | |  | tradno |
| U2 | Community health worker |  | |  | chwno |
| U3 | Nurse or midwife |  | |  | nursno |
| U4 | Pharmacist |  | |  | pharmno |
| U5 | General medical doctor |  | |  | gendocno |
| U6 | Non-psychiatric specialist |  | |  | specdocno |
| U7 | Psychiatrist |  | |  | psyno |
| U8 | Psychologist |  | |  | psychno |
| U9 | Counsellor |  | |  | counsno |
| U10 | Psychiatric nurse |  | |  | psynurseno |
| U11 | Mental Health cell coordinator |  | |  | mhcellno |
| U12 | Psychiatric clinical officer |  | |  | pco |
| U13 | Social worker |  | |  | swno |
| U14 | Religious or spiritual advisor |  | |  | relno |
| U15 | Other health worker |  | |  | omhno |
| Instruction:  “*In the last 3 months you have visited health providers [total visits above] time(s). I will now ask you questions about each of these visits. We will start with your most recent visit.”* | | | | | |
| U16 | For your [#th] outpatient visit, who did you see? | Traditional healer/spiritualist/herbalist | | 1 | opdwhoo_# |
|  |  | Community health worker | | 2 |  |
|  |  | Nurse or midwife | | 3 |  |
|  |  | Pharmacist | | 4 |  |
|  |  | General medical doctor | | 5 |  |
|  |  | Non-psychiatric specialist | | 6 |  |
|  |  | Psychiatrist | | 7 |  |
|  |  | Counsellor | | 8 |  |
|  |  | Psychiatric nurse | | 9 |  |
|  |  | Social worker | | 10 |  |
|  |  | Religious or spiritual advisor | | 11 |  |
|  |  | Psychologist | | 12 |  |
|  |  | Mental Health cell coordinator | | 13 |  |
|  |  | Psychiatric clinical officer | | 14 |  |
|  |  | Other health worker  [🡪 answer U17] | | 77 |  |
| U17 | Other provider [Specify] |  | |  | opdwhoo_# |
| U18 | Other provider [recoded] | [*Clean version of U17*] | |  | opdwho_#_rec |
| U18 | Where did you see the [provider in U16]? | Own home | | 1 | opdwhere_# |
|  |  | Local health centre | | 2 |  |
|  |  | Private office | | 3 |  |
|  |  | Hospital outpatient | | 4 |  |
|  |  | Provider’s home | | 6 |  |
| U19 | Why did you see the [provider in P16]? | Infectious disease (e.g. HIV, malaria, TB) | | 1 | opdwhy_# |
|  |  | Maternal / perinatal condition | | 2 |  |
|  |  | Acute condition (e.g. flu, cough) | | 3 |  |
|  |  | Injury | | 4 |  |
|  |  | Sleep problems | | 5 |  |
|  |  | Depression or anxiety | | 6 |  |
|  |  | Alcohol problems | | 7 |  |
|  |  | Other mental health problems | | 8 |  |
|  |  | Other chronic disease (e.g. heart, diabetes) | | 9 |  |
|  |  | Psychosis | | 10 |  |
|  |  | Epilepsy | | 11 |  |
|  |  | Other [🡪 answer U20] | | 77 |  |
|  |  | Don’t know | | 88 |  |
| U20 | Other condition [specify] |  | |  | opdwhyo_# |
| U21 | Other condition [recoded] | [*Clean version of U20*] | |  | opdwhyo_#_rec |
| Instruction:  “*What were the main features of the visit to the [provider in U16]?”* [Select up to three features] | | | | | |
| U22 | Assessment and/or diagnosis | No | | 0 | opdfeat_#  _assessment |
|  |  | Yes | | 1 |  |
| U23 | Drug prescription (drug for condition listed in the U19) | No | | 0 | opdfeat_#  _drugvisit |
|  |  | Yes | | 1 |  |
| U24 | Drug prescription (for other condition) | No | | 0 | opdfeat_#  _drugo |
|  |  | Yes | | 1 |  |
| U25 | Psychosocial support/care | No | | 0 | opdfeat_#  _psychsupport |
|  |  | Yes [complete U48-U50] | | 1 |  |
| U26 | Follow-up visit | No | | 0 | opdfeat_#  _followup |
|  |  | Yes | | 1 |  |
| U27 | Referral (to other provider) | No | | 0 | opdfeat_#  _referral |
|  |  | Yes [complete U34-U47] | | 1 |  |
| U28 | Prayer | No | | 0 | opdfeat_#  _prayer |
|  |  | Yes | | 1 |  |
| U29 | Traditional/spiritual healing | No | | 0 | opdfeat_#  _spiritual |
|  |  | Yes | | 1 |  |
| U30 | Other | No | | 0 | opdfeat_#  _other |
|  |  | Yes [🡪 answer U32] | | 1 |  |
| U31 | Don’t know | No | | 0 | opdfeat_#  _dontknow |
|  |  | Yes | | 1 |  |
| U32 | Other feature [specify] |  | |  | opdfeato_# |
| U33 | Other feature [recoded] | [*Clean version of U32*] | |  | opdfeato_#_rec |
| Instruction:  [If respond yes to U27] “To whom did you get a referral?” | | | | | |
| U34 | Traditional healer/spiritualist/herbalist | | No | 0 | opdreferral_#  _tradhealer |
|  |  |  | Yes | 1 |  |
| U35 | Community health worker | | No | 0 | opdreferral_#  _chw |
|  |  |  | Yes | 1 |  |
| U36 | Nurse or midwife | | No | 0 | opdreferral_#  _nurse |
|  |  |  | Yes | 1 |  |
| U37 | Pharmacist | | No | 0 | opdreferral_#  _pharmacist |
|  |  |  | Yes | 1 |  |
| U38 | General medical doctor | | No | 0 | opdreferral_#  _gp |
|  |  |  | Yes | 1 |  |
| U39 | Non-psychiatric specialist | | No | 0 | opdreferral_#  _specialist |
|  |  |  | Yes | 1 |  |
| U40 | Psychiatrist | | No | 0 | opdreferral_#  _psychiatrist |
|  |  |  | Yes | 1 |  |
| U41 | Counsellor | | No | 0 | opdreferral_#  _counsellor |
|  |  |  | Yes | 1 |  |
| U42 | Psychiatric nurse | | No | 0 | opdreferral_#  _psychnurse |
|  |  |  | Yes | 1 |  |
| U43 | Social worker | | No | 0 | opdreferral_#  _sw |
|  |  |  | Yes | 1 |  |
| U44 | Religious or spiritual advisor | | No | 0 | opdreferral_#  _spiritleader |
|  |  |  | Yes | 1 |  |
| U45 | Group counselling | | No | 0 | opdreferral_#  _gpcouns |
|  |  |  | Yes | 1 |  |
| U46 | Other health worker | | No | 0 | opdreferral_#  _other |
|  |  |  | Yes | 1 |  |
| U47 | Don’t know | | No | 0 | opdreferral_#  _dontknow |
|  |  |  | Yes | 1 |  |
| U48 | [Only if answer ‘yes’ to U25]  You mentioned that you received psychosocial care [Brief narrative about the content of the psychosocial treatment] | |  |  | opdnarrative_# |
| U49 | [Only if answer ‘yes’ to U25]  Brief narrative [recoded] | | [*Clean version of U48*] |  | opdnarrative_#  _rec |
| U50 | [Only if answer ‘yes’ to U25]  How many sessions of this kind will you complete in total? | |  |  | opdnarrative  num_# |
| U51 | How satisfied are you with the treatment you received from [provider in U16]? | | Very satisfied | 1 | opdsatis_# |
|  |  |  | Satisfied | 2 |  |
|  |  |  | Neither satisfied or dissatisfied | 3 |  |
|  |  |  | Dissatisfied | 4 |  |
|  |  |  | Very dissatisfied | 5 |  |
|  |  |  | Don’t know | 88 |  |
| U52 | How long did it take you to travel to the [provider in U16]? [Enter number of minutes, one direction] | |  |  | opdtravel_# |
| U53 | How long did you wait for your consultation with the [provider in U16]? [Enter number of minutes] | |  |  | opdwait_# |
| U54 | How long was the consultation with the [provider in U16]? (excluding waiting time)? [Enter number of minutes] | |  |  | opdlong_# |
| U55 | How much did you, your family or friends have to pay for the consultation with the [provider in U16]? (local currency units) | |  |  | opdfees_# |
| U56 | How much did you, your family or friends have to pay for travel? (local currency units) | |  |  | opdfare_# |

**Table S5.** Unit costs used in costing health service delivery

|  | ***Unit*** | **Source** | **Adjusted** |
| --- | --- | --- | --- |
|  |  | (2015 USD) | (2020 Int$) |
| **Inpatient care** | |  |  |
| Private hospital | *Day* | $5.26 | $24.16 |
| Public / NGO hospital | *Day* | $4.08 | $18.74 |
|  |  |  |  |
| **Outpatient care** | |  |  |
| Private hospital | *Visit* | $2.08 | $9.55 |
| Public / NGO hospital | *Visit* | $1.47 | $6.75 |
|  |  |  |  |
| Specialist doctor / psychiatrist | | |  |
| Consultation in private hospital | *Minute* | $0.31 | $1.42 |
| Consultation in public / NGO hospital | *Minute* | $0.22 | $1.01 |
|  |  |  |  |
| General doctor / medical officer | | |  |
| Consultation in private hospital | *Minute* | $0.26 | $1.19 |
| Consultation in public / NGO hospital | *Minute* | $0.18 | $0.83 |
| Consultation outside hospital (e.g. health centre) | *Minute* | $0.05 | $0.23 |
|  |  |  |  |
| Nurse / midwife / psychiatric nurse | | |  |
| Consultation in private hospital | *Minute* | $0.08 | $0.37 |
| Consultation in public / NGO hospital | *Minute* | $0.06 | $0.28 |
| Consultation outside hospital (e.g. health centre) | *Minute* | $0.03 | $0.14 |
|  |  |  |  |
| Counsellor / social worker | | |  |
| Consultation in private hospital | *Minute* | $0.08 | $0.37 |
| Consultation in public / NGO hospital | *Minute* | $0.06 | $0.28 |
| Consultation outside hospital (e.g. health centre) | *Minute* | $0.03 | $0.14 |
|  |  |  |  |
| Community health worker | |  |  |
| Consultation outside hospital (e.g. health centre) | *Minute* | $0.00 | $0.02 |
|  |  |  |  |
| **Psychological treatment** | |  |  |
| Individual session | *Minute* | $0.03 | $0.14 |
|  |  |  |  |
| **Psychotropic medication** | |  |  |
| Anti-psychotic medication | | |  |
| Chlopromazine (100 mg) | *Tablet* | $0.02 | $0.09 |
| Haloperidol (5 mg) | *Tablet* | $0.03 | $0.14 |
| Risperidone (2 mg) | *Tablet* | $0.03 | $0.14 |
| Fluphenazine (25 mg) | *Tablet* | $0.81 | $3.72 |
| Mood-stabliser medication | | |  |
| Carbemazepine (200 mg) | *Tablet* | $0.02 | $0.09 |
| Valproate (1500 mg) | *Tablet* | $0.20 | $0.93 |
| Anti-depressant medication | | |  |
| Amytryptyline (50 mg) | *Tablet* | $0.06 | $0.27 |
| Imipramine (100 mg) | *Tablet* | $0.02 | $0.07 |
| Fluoxetine (20 mg) | *Tablet* | $0.07 | $0.32 |
| Anti-epileptic medication | | |  |
| Phenobarbitone (100 mg) | *Tablet* | $0.03 | $0.14 |
| Sodium valproate (200 mg) | *Tablet* | $0.07 | $0.32 |
| Phenytoin (100 mg) | *Tablet* | $0.04 | $0.18 |
| Other medications | |  |  |
| Thiamine (100 mg) | *Tablet* | $0.07 | $0.32 |
| Biperiden (2 mg) | *Tablet* | $0.04 | $0.18 |
| Source: Chisholm et al., 2020 | | | |

**Supplementary figures**

**Figure S1.** CONSORT Flow Diagram

Assessed for eligibility (n= 2044)

Excluded (n= 1924)

♦  Did not meet inclusion criteria (n= 1907)

♦  Declined to participate (n= 17)

Analysed (n= 60)
♦ Missing data multiply imputed (n= 9)

♦ Cost outlier multiply imputed (n= 1)

Lost to follow up (n= 9)

♦ Moved away (n= 7)

♦ Refused (n= 2)

Allocated to standard care (n= 60)

♦ Received allocated intervention (n= 60)

Lost to follow up (n= 14)

♦ Moved away (n= 6)

♦ Refused (n= 6)

♦ Kept cancelling (n= 1)

♦ Hospitalized (n= 1)

Allocated to care + HAP (n= 60)

♦ Received allocated intervention (n= 52)

♦ Did not receive allocated intervention (n= 8)

Analysed (n= 60)
♦ Missing data multiply imputed (n= 14)

## Allocation

## Analysis

## Follow-Up

Randomized (n= 120)

## Enrollment

**Figure S3.** Cost-effectiveness acceptability curve.

GDP Gross domestic product, I$ international dollars

**Figure S2.** Nonparametric uncertainty analysis, per-participant approach. GDP pc Gross domestic product per capita, I$ international dollars
